# Instruction consisting of a rule and set of examples and nonexamples reliably teaches concepts

**DOI:** 10.1002/jeab.70061

**Published:** 2025-10-23

**Authors:** Catherine L. Williams, Jennifer C. Roop

**Affiliations:** ^1^ Department of Psychology University of North Carolina Wilmington Wilmington NC USA

**Keywords:** concepts, discrimination, generalization, instruction, instructional design

## Abstract

Conceptual learning is discrimination between new examples and nonexamples and generalization to new examples. Conceptual learning can be demonstrated after practice with differential reinforcement of the correct response and is influenced by procedural variables during practice. However, less research has been done identifying an ideal structure for instruction (rules), which is likely a typical teaching format for learners with more advanced verbal repertoires. We developed a laboratory analog of conceptual instruction to evaluate conceptual learning following instruction made up of a rule describing the key features of the concept and examples and nonexamples that were carefully selected to demonstrate these rules. We also evaluated the efficacy of this instruction when it preceded or followed practice with feedback about accuracy but no rule presentation. All participants completed instruction and practice. The specific instructional sequence was completed before practice during Experiment 1 and after practice during Experiment 2. This instructional sequence reliably and rapidly resulted in concept learning regardless of whether it was completed before or after practice. Practice alone never produced conceptual learning within the duration of the session and was not necessary to produce conceptual learning. Instructors should evaluate the efficacy of this instructional sequence to teach concepts.

The first step to designing effective instruction is categorizing the desired learning outcomes to directly inform what teaching approach is needed (Layng, 2014; Wiggins & McTighe, 2005). Tiemann and Markle (1990, most recently revised by Layng, 2014) provide one approach to categorizing learning. Their approach differs from others in that each category is related to the procedures needed to produce this learning (expanded on in Markle, 1990). Tiemann and Markle's categorization consists of a three‐by‐three matrix producing nine categories of learning. One dimension of the matrix is the complexity of the skill: differentiated relations (psychomotor), discriminative relations (simple cognitive), and extended relations (complex cognitive). Extended relations are those that require the learner to expand beyond what they are directly taught to generate something new. The other dimension of the matrix is the size of the behavioral unit: basic unit, linked units, or combined units. In the case of extended relations, basic units require the acquisition of a single association (i.e., conceptual learning), linked units require the acquisition of principles that describe the relation between multiple associations (i.e., rule application), and combined units require the combination of strategies to address a new situation (i.e., problem solving). All are common desired learning outcomes, but each requires different instructional approaches.

The basic unit of extended relations is conceptual learning. Conceptual learning occurs when learners can identify new examples of a concept (i.e., *generalization*) and tell the difference between what is and is not a new example (i.e., *discrimination*; Fleming & Levie, 1993; Markle & Tiemann, 1970; Mechner, 1965; Woolley & Tennyson, 1972; Zentall et al., 2002). For example, when teaching the concept of “triangle,” learners can demonstrate conceptual learning by selecting the “triangle” they have never seen before (i.e., generalization) among many new shapes (i.e., discrimination). Generalization to and discrimination between novel stimuli may be a product of different kinds of stimulus and functional relations. We will focus on concepts that are defined by shared physical features (e.g., conjunctive concept; Layng, 2019; e.g., natural concepts; Herrnstein et al., 1976). Concepts defined by shared physical features describe many learning outcomes relevant to learners of all ages from teaching “triangle” to early learners to teaching “conjoint schedules” in postsecondary education and beyond. Therefore, identifying methods that reliably and efficiently produce conceptual learning will support a wide range of clinical, educational, and occupational service providers.

Typically, concepts are taught using some combination of examples, nonexamples, and rules. Examples, nonexamples, and rules can be incorporated in two general ways: *instruction*, which does not require a learner response, and *practice* (i.e., *active learning*; Prince, 2004; i.e., *differential reinforcement*), which requires a learner response and feedback on the accuracy of that response. However, conceptual learning can occur in the absence of instruction through differential reinforcement (e.g., Eikeseth et al., 2014; Herrnstein et al., 1976), so learners who do not have the prerequisite skills to apply multiple rules can still learn concepts. Differential reinforcement to produce conceptual learning has been arranged using at least three general procedures. Go/no‐go procedures involve presenting a stimulus, measuring responding, and reinforcing responding in the presence of some stimuli and not others. Differences in responding between stimuli are measured to evaluate conceptual learning (e.g., Reynolds, 1961). Simultaneous discrimination procedures involve presenting multiple stimuli at the same time, measuring selection, and reinforcing selection to some stimuli but not others. Selection of stimuli without a history of reinforcement is measured to evaluate conceptual learning (e.g., Mackintosh et al., 1968). Conditional discrimination procedures are similar to simultaneous matching procedures except that a discriminative stimulus is also presented (either at the same time as or prior to the other stimuli) that indicates which response will be reinforced (e.g., Maki & Leuin, 1972).

Using these different procedural arrangements, previous research has evaluated how the selection and presentation of stimuli during practice affect conceptual learning, such as beginning with more disparate stimuli to promote discrimination (e.g., Swets, 1959) including similar stimuli during training (Williams et al., 2025), ensuring that the relation between response and consequence is salient (Fisher et al., 2014), and using examples that differ in as many unimportant ways as possible (Layng, 2019). The number of stimuli introduced in a conceptual set also appears to affect conceptual learning, but more research is needed to specify the variables that determine the direction of this effect (Bodily et al., 2008; Kodak et al., 2020; Lazarowski et al., 2019; Nakamura et al., 2009; Vladescu et al., 2021; Williams et al., 2024). However, these manipulations were made in the absence of instructions about how to select the correct response.

It is possible that when the learner demonstrates the prerequisite skills to follow multiple rules, providing instruction may wash out the effects of practice variables. Although there is research comparing variables on instructional procedures and practice procedural variables in isolation, there is less comparing sequences of instruction and practice or evaluating the efficacy of one over the other. Instruction and practice are separate components that each promote learning (Danforth et al., 1990). Practice is focused on the relation between a response and its accuracy, whereby a correct response is differentially reinforced (i.e., contingency‐shaped behavior). In contrast, instruction is an antecedent strategy that should increase the likelihood of a correct response when the option to respond is provided (i.e., rule‐governed behavior).

Previous research comparing contingency‐shaped and rule‐governed responding has demonstrated that rules will govern behavior until the point at which responding contacts a consequence conflicting with the rule (e.g., Galizio, 1979). In the case of classroom instruction, instructions should present rules consistent with the contingencies, but the kinds of contingencies differ from most basic research. Previous research has evaluated rule‐governed behavior when rules were used to teach a chain of simple responses. For example, Danforth et al. (1990) evaluated how undergraduate students learned a 12‐component chain following differential reinforcement or rules. They found that either could be used to teach the chain but that the rules taught it faster and with fewer errors than differential reinforcement. Okouchi (2023) reinforced a chain that was a sequence of two responses (out of eight options), and efficient responding was also acquired by the end of the experiment. However, they noted that fewer of the initial participants obtained reinforcers for this response chain than in an experiment conducting a similar evaluation using a multiple fixed‐ratio, differential‐reinforcement‐of‐low‐rates schedule (Okouchi, 2022). They theorized that rules may work differently depending on the contingencies. Instruction for conceptual learning could be conceptualized as teaching a sequence of discriminations, where each discrimination is based on a defining feature of the concept. Although previous research supports the potential for rules to expedite learning, the effects of rules on conceptual learning have yet to be experimentally evaluated. In addition, it remains unclear how the rules for conceptual learning should be selected.

Evans et al. (1962; as described in Markle, 1990) provided a system to use to describe the contents of instruction. Instruction is divided into rules (RUL) and examples (EG) leading to a *RULEG* system. To design RULEG instruction for concepts, one must first conduct a concept analysis (Johnson & Bulla, 2021; Tiemann & Markle, 1990). A concept analysis is conducted to identify what features *must* be present for a stimulus to be an example of the concept (i.e., *must‐have features*) and what features *can* be present without altering whether a stimulus is an example (i.e., *can‐have features*). The RULEG instruction begins by stating the must‐have features and can‐have features as rules. Next, examples follow to demonstrate these rules. The examples should consist of a pair of examples that differ according to all can‐have features (i.e., divergent examples). This pair of examples promotes generalization between examples. These examples are followed by a set of nonexamples that each lack exactly one must‐have feature but have identical can‐have features. The nonexamples promote discrimination between examples and nonexamples (Williams et al., 2025). This combination of examples and nonexamples is referred to as a *rational set* (Layng, 2019; Tiemann & Markle, 1990).

To our knowledge, no research has evaluated the use of RULEG instruction to teach concepts in the absence of practice. We developed a translational research approach to evaluate RULEG instruction. We taught arbitrary concepts to control for previous knowledge and did so out of a classroom setting to minimize student exposure to lessons that may not be effective. Demonstrating the efficacy of this instruction will provide a tool for skill builders to use to teach concepts quickly and efficiently. Therefore, the primary objective of this experiment was to evaluate the efficacy of RULEG instruction using an arbitrary concept taught in a controlled laboratory setting.

## EXPERIMENT 1

### Method

#### Participants

Participants were recruited through an online system provided by the university. Each participant signed up for one session that lasted up to 3 hr. Anyone with access to this system and who was at least 18 years of age was eligible to participate. One participant was excluded due to a researcher entering an incorrect parameter value while setting up the program, and another was excluded due to a programming error that caused the program to provide only one pretest block. Of the four participants included, three participants were female and one was male; all were white. Their mean age was 18.75 (range 18 to 19) years. Before starting the experiment, each participant signed an informed consent document through a process approved by the university's Institutional Review Board for the Protection of Human Subjects.

They earned or lost $0.10 for each correct or incorrect response, respectively. The participants were given the money they earned rounded up to the nearest $5 increment immediately after the experiment in the form of an Amazon gift card. A 25% bonus was added to the value of the cards if the participants completed the entire experiment. Earnings were adjusted to the nearest value that fell within the range of $10–20 per hour to ensure the wage was fair but not coercive. Three of the participants earned $25, and one (P1) earned $20.

#### Setting and apparatus

Each participant worked alone in a small area separated from the rest of the university laboratory space by walls on three sides and a foldable barrier on the fourth and equipped with only a chair and table. Before each session, the participant left all personal belongings in a separate secure area. The experimental task was presented using a custom Visual Basic program on a Dell Latitude 7320 Detachable tablet with a touch screen (33.02‐cm display; 1920 × 1280 pixel resolution). No keyboard or mouse was available during the session. Sounds were played through headphones that were connected to the tablet; the volume setting was 50% and participants wore noise‐canceling headphones plugged into the computer. Each trial consisted of the appearance of four stimuli on the screen, the selection of a stimulus, and the delivery of feedback. All responses were made by touching the screen.

At the start of each trial, a white rectangular button (116 × 42 pixels, approximately 3.0 × 1.1 cm) appeared on the screen with the word “START” printed on it in 20‐point type. When a participant touched the button, it disappeared and four stimuli appeared (one example and three nonexamples). Each stimulus was 250 × 250 pixels (approximately 6.6 × 6.6 cm) and located 0.5 cm away from the two sides of the screen that defined a corner.

The stimuli remained on the screen until the participant selected one by touching it (i.e., simultaneous discrimination procedure). When a stimulus was selected, all stimuli disappeared and, depending on the selection and experimental condition, positive, negative, or neutral feedback (details provided in the *Tests* and *Practice* sections) was presented in the center of the screen. All forms of feedback ended after 1 s, followed by a 1‐s intertrial interval during which a blank, white screen was shown.

If a participant did not select a response within 1 min, all stimuli and text on the screen disappeared and a message appeared in the middle of the screen that said, “Please respond as quickly as you can” with a button below it containing the word “OK.” When “OK” was selected, the same stimuli and text reappeared, and the trial timer resumed. All participants in both experiments responded to all trials within 1 min, so they never received this message. Unless noted otherwise, all text displayed by the program was in black, Microsoft Sans Serif 24‐point font.

The tests and practice consisted of blocks of 24 trials. Blocks of 24 trials were selected because they allowed stimuli to be shown an equal number of times and equally in each location. There were no indicators of when one block ended and another started unless it was time for a break or the beginning of a test. In addition to breaks scheduled at particular points during the experiment (described in the Procedure), the participant got a break after finishing any block after more than 45 min elapsed from the last break.

#### Experimental design

To assess the relative roles of the instruction and practice components, all participants completed a pretest, RULEG instruction, midtest, practice, and posttest in that order.

#### Experimental stimuli

**FIGURE 1:**
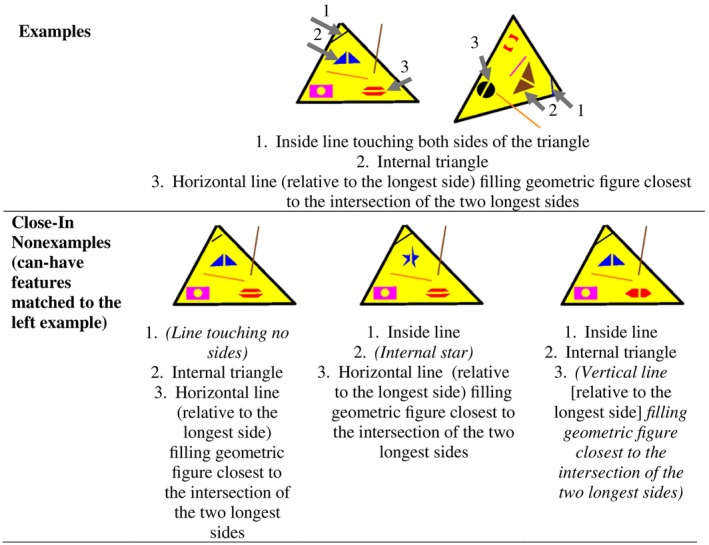
Examples and close‐in nonexamples of the concept. Features in parentheses are replacement features. The colors of the features were changed across examples and nonexamples, but the triangle fill was always yellow. In the top (Examples) row, the gray arrows and numbers label each feature based on its number. They were not part of the stimuli presented during practice or testing.

We created arbitrary stimuli to represent a concept that might be taught in a class and that is defined by must‐have features and can‐have features. The use of an arbitrary concept ensured that participants did not have any history of reinforcement related to the concept being taught. This approach addresses previous research demonstrating that effects of instruction and practice differ based on the level of prior knowledge (e.g., Danforth et al., 1990; He et al., 2025). Each stimulus had 12 features: three lines, three geometric objects, three geometric‐object fills, the color and size of each of the preceding nine features, and the degree of rotation of the figure. Three of these features were must‐have features of each concept we taught. To be an example of the concept, the stimulus had to have all three must‐have features. A stimulus with just two must‐have features was classified as a close‐in nonexample, and a stimulus with no must‐have features was classified as a far‐out nonexample. The must‐have features of the concept were (1) a line connecting two sides of a triangle without going outside of the triangle, (2) a smaller triangle within the large triangle, and (3) a horizontal line filling the geometric figure closest to the intersection of the two longest sides of the triangle. The remaining nine features were all can‐have features. Figure 1 provides divergent examples and three close‐in nonexamples of the concept we taught. We created the stimuli with Omber Version 1.41 (Wobastic Software Inc., 2019). These stimuli are available in Supplemental Materials.

#### Procedure

The number of trials, trial arrangements, and stimuli are summarized in Table 1.

**TABLE 1:** Overview of phases and stimuli.

Phase	# Trials per block	# Trials with practiced examples	# Trials with practiced close‐in nonexamples	# Trials with unpracticed close‐in nonexamples	# Trials with unpracticed far‐out nonexamples	# Trials with unpracticed examples
Tests	24	12 (6)	12 (18)	6 (18)	6 (18)	12 (12)
Practice	24	24 (6)	24 (18)	0	0	0

*Note*: The parenthetical values indicate the number of distinct stimuli of that type.

##### Preexperimental procedures

When the potential participant arrived, they sat at a table near the entryway while the researcher read the consent form to them. The consent form included the following description of the research task and purpose:

We hope to figure out how people learn the best so we can help others know how to teach the best. We want to find out the best: Way to teach so that people can apply what they learn; sequence of instruction; format of instruction; difficulty of instruction; indicator of how well people learned the material. We will ask you to: select an answer from a list of possible responses on a computer and learn how to select the correct response; select an answer without telling you whether your answer is correct (similar to a test); view instructions that may help you select the correct response; share strategies and feelings about how you selected your responses; complete this process multiple times to learn different things in different ways. You may be taught something real or something made up to conduct research. We may dismiss you from the research if you already know what we want to teach you.

If the individual consented, they were told to leave all their belongings in the consent area then escorted to the research setting. The researcher read the following instructions:

Please put the headphones on and read the instructions on the screen. Please leave the headphones on at all times, as there will be audio instructions at some point. If you need to adjust the volume, you can do so with these buttons (show the buttons). When you are ready to begin, press start. The program will tell you when the session is over. At this time, please knock on the wall to let me know you're done.

The only text on the screen was “Start.” If at any point the participant asked the researcher questions, they answered using language directly from the consent form, script, or said “the instructions on the screen are the only ones you will get.” Throughout the remainder of the experiment, the only interactions between the researcher and participant were to let them know they could access their belongings and where to go during break. The researcher also repeated the second part of the instructions provided previously following each break.

##### Tests

The pretest, midtest, and posttest contained identical trials in a random order. Tests consisted of three blocks of 24 trials containing a total of 72 unique stimuli. Each block contained three trial types. Twelve trials were directly from practice (one example and three close‐in nonexamples using all 24 stimuli from practice twice). The other 12 trials contained stimuli not used during practice, so participants never received feedback on the accuracy of their responses in the presence of these stimuli. Six of these trials contained one example and three close‐in nonexamples from a set of six examples and 18 close‐in nonexamples. Six trials with unpracticed stimuli contained one example and three far‐out nonexamples out of a set of six examples and 18 far‐out nonexamples. Each unpracticed example and nonexample was shown once per block, but which examples and nonexamples were shown together was randomized at the start of each block. A trial example and all test stimuli are available in the Supplemental Materials.

All responses were followed by neutral feedback. The following instructions appeared in text at the top, center of the screen before the first trial of the test began:

You will be shown four images at a time. Your job is to select an image you think is right. For now, you will not be told whether your selection is right or wrong. You will earn $0.10 for each correct answer and lose the same amount for each incorrect response. When you are done, a message will appear telling you how much you earned and giving you instructions for the next part of the study, when you will learn how to identify the correct answers or you may have finished the study.

The start button (identical to the one that also appeared at the start of each trial) was placed directly below this text. The test then began when the start button was touched. All responses were followed by neutral feedback. Neutral feedback consisted of text that said “Selected.” No auditory stimulus was played, and the total amount of money earned was not shown.

##### RULEG instruction

Immediately after the preceding test, the following instructions appeared:

You will be shown a video telling you how to identify the correct answer. Please pay close attention to the video. Alert the researcher if you cannot hear the instructions. After the instructions, you will be shown four images at a time. Your job is to select an image you think is right. For now, you will not be told whether your selection is right or wrong. You will earn $0.10 for each correct answer and lose the same amount for each incorrect response. When you are done, a message will appear telling you how much you earned and giving you instructions for the next part of the study, when you will learn how to identify the correct answers or you may have finished the study.

After clicking next, a video filled the screen to provide the instruction. The video was recorded using PowerPoint and consisted of spoken audio with subtitles over seven slides that provided RULEG instruction (Evans et al., 1962; Markle, 1990). The rule was presented in the first slide, which contained three triangles. Each displayed one must‐have feature and had no other features, and the narration described each of the must‐have features. The remaining slides presented a minimum rational set of examples and nonexamples. The second slide contained an example, and the narration and animation described and identified each must‐have feature. The third slide contained a divergent example, meaning all of the can‐have features were different from the first example. Again, the narration and animation described and identified each must‐have feature. Slides 4–6 each showed the example next to a close‐in nonexample. The same example was used for all three slides. The nonexample differed from the example only in that it lacked one of the three must‐have features. Across the three slides, each lacked a different must‐have feature. The narration and animation described and identified the missing must‐have feature. The final slide showed the same example and all three nonexamples from the previous four slides, one in each corner (similar to the trials). The narration and animation again pointed out the example and the feature missing from each nonexample. Participants could not stop, skip, or rewind the video. Instruction lasted 2 min 33 s and is available in the Supplemental Material.

##### Practice

Participants started with $14.40 (the equivalent of getting all questions correct for six blocks) at the beginning of practice so that all participants were equally motivated to respond correctly, although they were not told that this value did not change based on their pretest and midtest responding. Immediately after the preceding test, the following instructions were shown before the first practice trial:

You will be shown four images at a time. You will earn $0.10 each time you select a correct image and lose the same amount each time you select an incorrect image. When you select an image, you will be told whether your selection is right or wrong. When you are done, a message will appear on the center of the screen telling you to go get the researcher.

After clicking next, the first practice trial appeared. Practice consisted of blocks of 24 trials. Each trial contained one example and three close‐in nonexamples (out of the same set of six examples and 18 close‐in nonexamples used for 12 of the test trials). This number of stimuli and use of examples and close‐in nonexamples were used based on the findings of Williams et al. (2025) and recommendations of Tiemann and Markle (1990, see also Johnson & Bulla, 2021).

When the participant touched an example (correct response) or a nonexample was selected (incorrect response), positive or negative feedback was provided, respectively. Positive feedback consisted of text that said “Right! + $0.10” and a concurrent trumpet fanfare played for 1 s. During the fanfare, the total amount of money earned so far in the experiment was displayed for 0.25 s, disappeared for 0.25 s, and reappeared for 0.5 s with $0.10 added to the total. Negative feedback consisted of text that said “Wrong. ‐ $0.10” and a concurrent buzzer sound that was played for 1 s. During the buzzer, the total amount of money earned so far in the experiment was displayed for 0.25 s, disappeared for 0.25 s, and reappeared for 0.5 s with 0.10 subtracted from the total. The total could not go below $0. After an incorrect response, a correction trial was conducted with the same four stimuli as the previous trial, but the stimuli were randomly reassigned to the corners of the screen. Feedback identical to that described previously followed responses on correction trials. Correction trials were not counted as one of the 24 trials in a block, nor were they included in summary data analyses for that block. Underneath the feedback text, a button labeled “Next” had to be touched to continue to the next trial, thereby ensuring the participant's hand was positioned in the center of the screen and that all responses required the same effort. We used different auditory and visual stimuli for each consequence to ensure that it was salient and discriminable, even if the participant was not looking at the screen. We did not provide additional feedback on what made the response correct or incorrect or prompting such as may be provided in applied discrete‐trial arrangements (Lerman et al., 2016). Such feedback would introduce rules to the practice, thereby making it unclear which was responsible for changes in responding.

Practice ended after the participant completed three consecutive blocks with correct responses on at least 23 of 24 trials or after a maximum of eight blocks. Participants were given a 5‐min break after practice. After the break, they completed one more practice block to measure any change in responding due to the break before beginning the next test.

### Results and discussion

**FIGURE 2:**
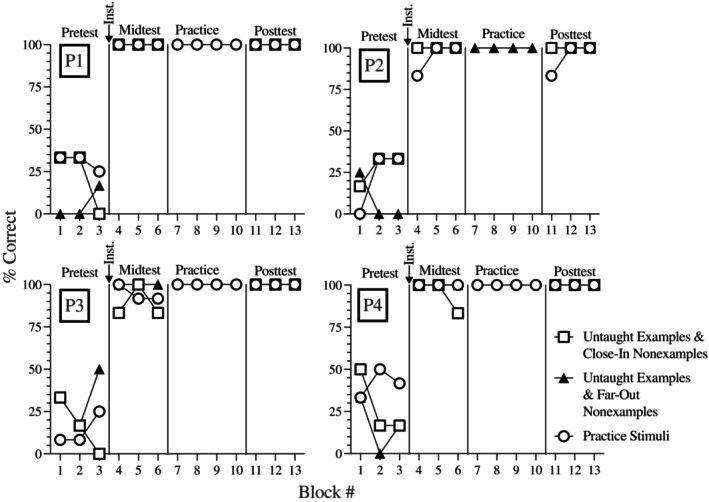
Accuracy for participants who completed RULEG instruction first. The participant number is indicated in the upper‐left corner of each graph. Each pretest, midtest, and posttest block contained six trials with untaught examples and close‐in nonexamples, six trials with untaught examples and far‐out nonexamples, and 12 trials with practice stimuli.

The graphs in Figure 2 show the percentage of correct responses on each block of 24 trials during the pretest, midtest, practice, and posttest in each condition for each participant. An arrow at the top of each graph depicts when the RULEG instruction occurred. During the pretest, accuracy was around chance levels. After just instruction, three participants (P1, P2, and P4) responded with perfect or near‐perfect (missed one trial out of the 72) accuracy during the midtest. The fourth participant (P3) made one or two errors during each midtest block on trials with examples and close‐in nonexamples. All four participants completed practice without making any errors. Median latencies to respond during practice decreased from a mean across participants of about 4.5 to 3.5 s across practice trials (trial‐by‐trial data are available on request to the first author). They also responded with perfect or near‐perfect accuracy on all posttest blocks.

After only RULEG instruction, three of the four participants demonstrated clear conceptual learning and the remaining participant (P3) made only four errors across the 72 test trials, showing substantial improvement from baseline. The results demonstrate that the RULEG instruction, which was provided in less than 3 min, was sufficient to teach a concept defined by three must‐have features.

Recall that the term concept is being used specifically to describe generalizing to novel examples and discriminating between novel examples and nonexamples. Although there has been some research comparing generalization or discrimination after various forms of practice (e.g., Fisher et al., 2014; Schnell et al., 2018; see Halbur et al., 2021 for an overview in the context of discrete trial training), we are not aware of research evaluating conceptual learning after RULEG instruction. The successful learning we observed after the RULEG instruction replicated previous research that rule‐governed behavior facilitates learning (Danforth et al., 1990; Okouchi, 2022). Our findings expand this research to conceptual learning and provide a clear framework for how rules should be presented for conceptual learning. When previous research did include instruction for conceptual learning, they did not typically report sufficient detail, such as reporting the concept analysis (Johnson & Bulla, 2021) and the relation between the examples and nonexamples given (Williams et al., 2025). Future research should evaluate conceptual learning with RULEG instruction for additional concepts and in classroom settings.

Participants also completed practice after instruction. Because they were all already responding accurately, this practice was not necessary. However, the question remains about whether practice alone would also teach the concept, so Experiment 2 was conducted to evaluate effects of practice before the RULEG instruction.

## EXPERIMENT 2

There are cross‐disciplinary conversations about whether practice or instruction should be the focus of classroom time. One hypothesis is that providing instruction first leads to better learning because it decreases the *cognitive load* (e.g., Kirschner et al., 2006; Mayer, 2001; Sweller, 1999). Cognitive load theory is based on the idea that as the working memory (conscious processes that occur between a stimulus and response) required for the task decreases, student learning increases. Decreases in cognitive load and working memory are assumed when better or faster learning is observed. Working memory can be decreased by minimizing the number of processes one needs to emit between the stimulus provided and the desired response. The number of processes can be minimized in different ways such as student prerequisite skills, helping students develop strategies to most directly get to the answer, or instructions (Kirschner et al., 2006). Instructions particularly reduce cognitive load when learners do not have previous experience with related strategies and concepts (He et al., 2025). Results produced through the application of cognitive load theory align well with the behavior‐analytic literature, as cognitive load is a reification of how a history of differential reinforcement related to prerequisite skills and rules facilitates the acquisition of component skills (e.g., Patterson et al., 2025).

Another hypothesis is that providing practice results in better learning because responding incorrectly uniquely enhances later learning (i.e., *productive failure*; e.g., Kapur, 2008; Kapur & Bielaczyc, 2012). When learners begin with practice, they use their prior knowledge to identify important features of the problem, organize them, and develop a method to solve them. These components parallel with the function of rules, examples, and nonexamples presented during RULEG instruction (Tiemann & Markle, 1990) but ask the learner to generate this information (i.e., self‐generated rules) during differential reinforcement rather than providing it directly through instruction.

Direct comparisons between the literature comparing cognitive load theory and productive failure in classroom instruction (e.g., He et al., 2025; Kapur & Bielaczyc, 2012) and behavior‐analytic literature on conceptual learning are challenging for at least two reasons, which could be considered in future research to bridge the gap. First, the behavior‐analytic literature typically provides details in the method section about exactly which stimuli, responses, and feedback were involved at what stages of instruction, whereas this level of detail is uncommon in the cognitive literature. Second, the cognitive and behavior‐analytic literatures categorize the hierarchies of learning outcomes in different ways. This means behavior‐analytic dependent variables often include multiple categories of learning that would be isolated by more cognitive approaches and vice versa.

Previous research has tested these theories by comparing learning between groups who experience instruction first and groups who experience practice first (Danforth, 1990; He et al., 2025; Kapur & Bielaczyc, 2012). However, none of these experiments evaluated conceptual learning. Different instruction is needed for different kinds of learning (Markle, 1990; Tiemann & Markle, 1990), so it is likely that the efficacy of instruction and practice differ between these skills. He et al. (2025) and Kapur and Bielaczyc (2012) evaluated problem solving and Danforth (1990) evaluated sequences (a subcategory of discriminative relations; Tiemann & Markle, 1990). In addition, among Danforth (1990), He et al. (2025), and Kapur and Bielaczyc (2012), only Danforth (1990) fully isolated instruction and practice. The other two studies had aspects of practice and instruction, as defined by the current experiments, in both conditions. Danforth et al. taught a sequence of responses (selecting a pattern of arbitrary stimuli, a discriminative relation) in a laboratory setting with undergraduate students. They compared the acquisition of this sequence when taught with various combinations of differential reinforcement and instructions. They found that all combinations resulted in learning the sequence but instruction alone did so most efficiently.

It is unclear how the purposes of instruction and practice to teach a concept interact when these steps are done in different orders. Because participants intentionally do not have a previous reinforcement history with the arbitrary concepts we use, we might expect instruction first to result in better acquisition, based on cognitive load theory and rulegoverned behavior. Practicing before instruction will likely result in more errors (given no prompting strategy or stimulus‐fading approaches are included; Sidman, 2010; Terrace, 1963a, 1963b; Touchette & Howard, 1984), but it may also alter the amount of practice completed, serve as a motivating operation for attending to instruction, or alter the effectiveness of instruction. Therefore, Experiment 2 evaluated conceptual learning when starting with practice to see whether practice alone produced conceptual learning. If practice alone was not sufficient, Experiment 2 also evaluated whether RULEG instruction following practice still effectively produces conceptual learning.

### Method

Participants were recruited the same way as in Experiment 1. Four participants completed the experiment. Three were female, and one was male; all were white. Their mean age was 18.75 (range 18 to 19) years. All participants earned $25.

All other procedures were identical to those for Experiment 1, except participants completed the practice first (between the pretest and midtest) and instruction second (between the midtest and posttest). All participants completed a pretest, practice, midtest, RULEG instruction, and posttest in that order.

### Results and discussion

**FIGURE 3:**
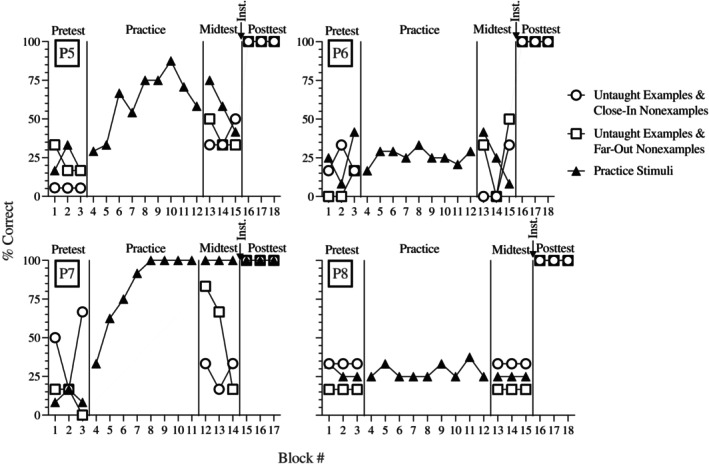
Accuracy for participants who completed practice first. The participant number is indicated in the upper‐left corner of each graph. Each pretest, midtest, and posttest block contained six trials with untaught examples and close‐in nonexamples, six trials with untaught examples and far‐out nonexamples, and 12 trials with practice stimuli.

Figure 3 displays data from participants in Experiment 2 (P5–8) in a manner like Experiment 1. Three of the four participants responded at near‐chance levels on all pretest trials. The exception was P7, who responded 44.4% correct on average during trials with novel examples and close‐in nonexamples. Three of four participants (P5, P6, and P8) completed the maximum number of practice trials (9) without ever meeting the mastery criterion. The median latencies to respond for P6 and P8 were around 1 s, which was lower than the latencies of the Experiment 1 participants when they responded accurately. This suggests these participants may have been guessing (i.e., responding under the control of the consequence of only the most recent response). Accuracy did not improve for P6 or P8 during practice or the midtest. The median latencies to respond for P5 and P7 were initially longer than the latencies of the Experiment 1 participants but decreased to latencies similar to those of the Experiment 1 participants across blocks (trial‐by‐trial data available on request to the first author). P5 responded more accurately on all midtest trial types than on the pretest, but accuracy remained below 75%. The fourth participant (P7) was the only one who completed practice first and met the mastery criterion. During the midtest, P7 maintained perfect accuracy on trials containing the practice stimuli. However, P7 responded less accurately on trials with untaught stimuli, especially those with examples and close‐in nonexamples, showing that the mastery criterion did not predict conceptual learning. After the RULEG instruction, all participants responded with perfect accuracy on all posttest trial types.

After only practice, none of the participants demonstrated conceptual learning. When RULEG instruction was provided after practice, it still effectively taught the concept to all participants. These results support providing RULEG instruction before practice to teach a concept. They also show that RULEG instruction remained effective even after a recent history of incorrect responses related to the concept.

## GENERAL DISCUSSION

Overall, all participants demonstrated conceptual learning after RULEG instruction and practice consisting of simultaneous discriminations between one example and three close‐in nonexamples using 24 total stimuli. Experiment 1 demonstrated that the RULEG instruction alone taught the concept, and Experiment 2 demonstrated that practice alone did not. Across all participants, regardless of their order, the RULEG instruction taught the concept. The RULEG instruction is a robust, efficient instructional approach that theoretically can be applied to teach any concept defined by shared features.

There are consistent differences between experiments, and conclusions drawn from these comparisons should be limited because participants were not randomly assigned to an experiment. Specifically, participants in Experiment 2 experienced practice differently from those in Experiment 1. When they completed practice first, it took longer and resulted in more errors. In addition, three out of the four participants in Experiment 2 did not meet the mastery criterion before the maximum number of practice blocks and two of those participants remained at pretest accuracy during the practice. All participants in Experiment 1 met mastery during practice.

The differences observed during the practice and midtest between experiments indicate that responding on these trials was likely under different control across experiments. During Experiment 1, participants were provided with accurate instructions prior to responding. Their responding afterward was likely occasioned by these rules (Danforth et al., 1990; Galizio, 1979; Okouchi, 2023). Therefore, during the midtest, accurate responding in the presence of untaught stimuli may not indicate stimulus generalization but the continued mediation of accurate responding based on these rules. However, during the practice and midtest in Experiment 2, responding may be occasioned by the history of differential reinforcement and/or self‐generated (although apparently inaccurate) rules, such as those categorized as problem solving (Miguel, 2018). During the posttest for both experiments, all participants had completed both instruction and practice. Responding during the posttest could theoretically be under joint control of the instructions provided experimentally (i.e., tacts of the relevant features) and the reinforcement history. It is possible that the order of these components might influence what occasioned posttest responding. Because Experiment 1 demonstrated conceptual learning under just the control resulting from instructions, it is possible that responding during the posttest was also just under the control of the instructions. Future research could collect verbal reports from participants about the degree to which they were applying the rules and what they learned from practice across phases of the experiment (e.g., Vie & Arntzen, 2017). Future research could also replicate previous research evaluating the effects of inaccurate instructions on responding (e.g., Danforth et al., 1990) but in the context of conceptual learning to evaluate whether instructions play a different role when the subject matter of interest involves generalization.

Although practice alone did not teach the concept, it also did not impede conceptual learning when instruction was provided later, which additionally supports that responding may be occasioned differently by instructions than by practice. This suggests that there is no reason to avoid starting with practice and designing for productive failure (e.g., Kapur, 2008) as long as instruction is provided later. However, participants who completed practice first were repeatedly exposed to punishment in the form of money loss (similar to point loss or negative feedback on a practice assignment) and repeatedly emitted incorrect responses. Beginning with instruction, thereby minimizing errors and the experience of punishment, is more in line with the Behavior Analyst Ethics Code (Behavior Analysis Certification Board, 2020). It is also a more efficient use of learner and instructor time. Future research should also include measures of participant preference for and social validity of these instruction and practice procedures.

In addition to intentionally programming practice first, as suggested in alignment with productive failure (e.g., Kapur, 2008), practice may also happen first because of learner fidelity errors. Even if instructors assign instruction before practice, if learners can complete practice before instruction, they may still choose to do so. When possible, instructors should design their lessons to prevent practice before instruction. Future research should evaluate the use of various approaches to ensure learners contact instructions prior to practice. However, when it is not possible to prevent practice before instruction, RULEG instruction may still teach the concept, even after extended practice.

Across these experiments, we evaluated only one form of instruction and one form of practice to teach conceptual learning. Future research should evaluate other instruction and practice procedures informed by those most commonly used to teach the desired skills to the population of interest or common errors during learning. Future research should compare this instruction with other strategies that are more typical (e.g., lectures supplied by the textbook), evaluate how typical instruction interacts with practice, and observe instructors to characterize what instruction and practice strategies are typical. This investigative process could also be repeated for the other types of learning, especially those in the extended relations category (Tiemann & Markle, 1990). These experiments provide a concrete method to design instructions for concepts that should be directly evaluated to enhance conceptual instruction in educational settings.

## AUTHOR CONTRIBUTIONS

JR contributed to the conceptualization, data curation, methodology and initial drafts of the manuscript. CW contributed to the conceptualization, data curation, investigation, methodology, project administration, software, supervision, visualization, and reviewed and edited drafts.

## CONFLICT OF INTEREST STATEMENT

We have no known conflict of interest to disclose.

## ETHICS APPROVAL

All procedures were approved by the University of North Carolina Wilmington Institutional Review Board.

## Supporting information


**Data S1:** Supporting Information.

## Data Availability

Data are available on request to the corresponding author.
