# Supplementary material for: Instruction consisting of a rule and set of examples and nonexamples reliably teaches concepts
Source: J Exp Anal Behav. 2025 Oct 23;124(3):e70061. doi: 10.1002/jeab.70061 (PMC12548561; doi:10.1002/jeab.70061)
Supplement: Supplementary file 1 — Data S1: Supporting Information. [file JEAB-124-0-s001.docx]

Trial Example

The three must-have features the concept we taught are: 1. Inside line; 2. Internal triangle; 3. Horizontal line filling geometric figure closest to the intersection of the two longest sides. Stimulus IV contains three must-have features, making it an example in the Close-In Nonexamples condition. Selecting Stimulus IV would be the only correct response. Stimuli I, II, and III contain a horizontal line through the bottom-right geometric figure and a triangle but lacks an inside line. Because Stimuli I, II, and III each lack one must-have feature, they are close-in nonexamples of the concept.


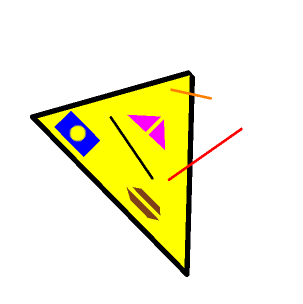

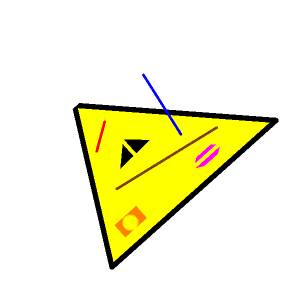
*
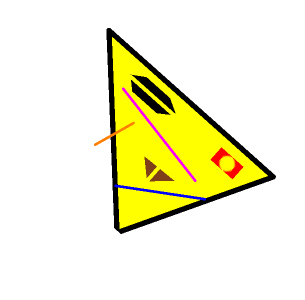
*
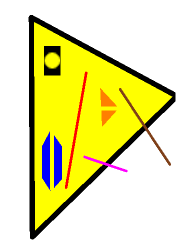


**I**

**II**

**IV**

**III**

**Practice Stimuli**

| Examples | | | |
| --- | --- | --- | --- |
| 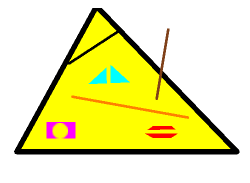 | 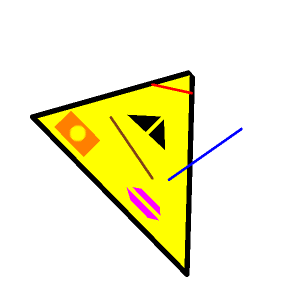 | 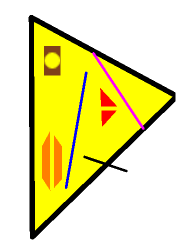 | |
| 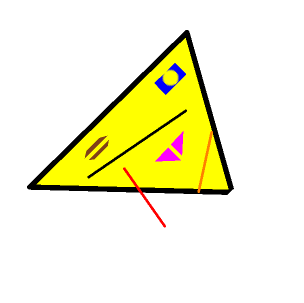 | 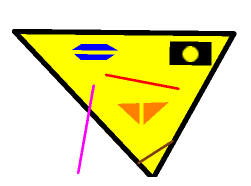 | 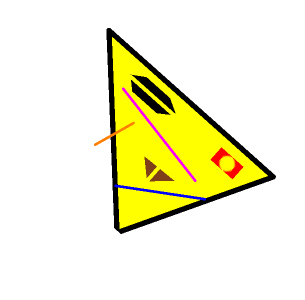 | |
| Close-In Nonexamples Lacking Inside Line | | | |
| 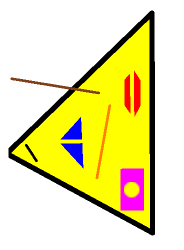 | 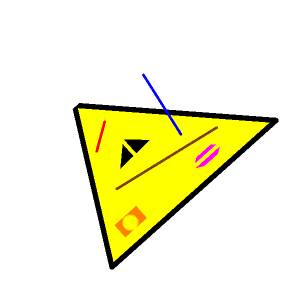 | 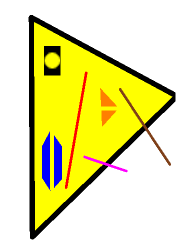 | |
| 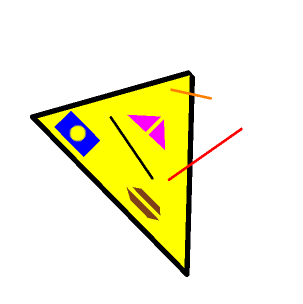 | 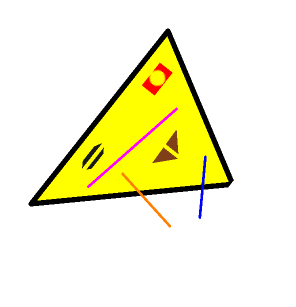 | 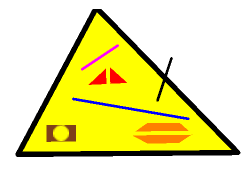 | |
| Close-In Nonexamples Lacking Internal Triangle | | | |
| 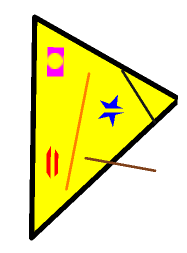 | 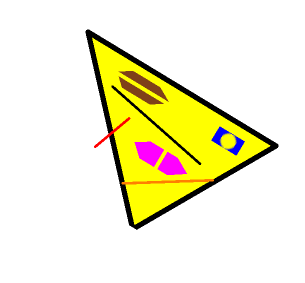 | 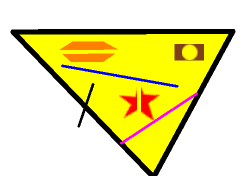 | |
| 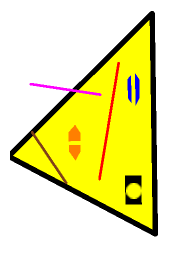 | 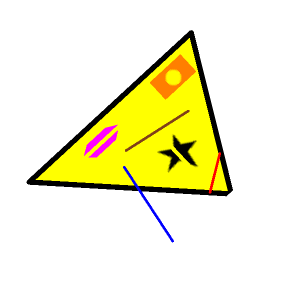 | 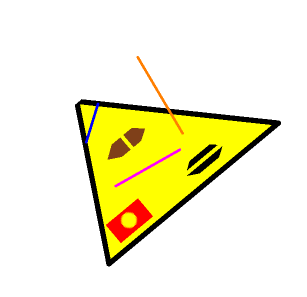 | |
| Close-In Nonexamples Lacking Horizontal Line Filling Geometric Figure Closest To The Intersection Of The Two Longest Sides | | | |
| 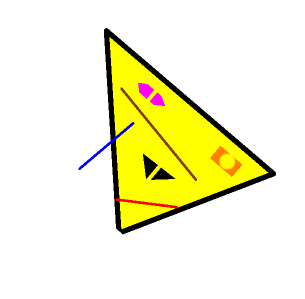 | 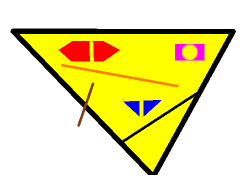 | | 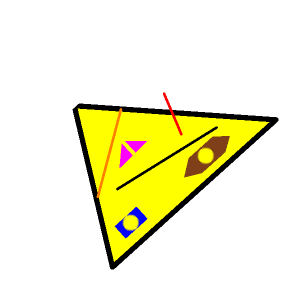 |
| 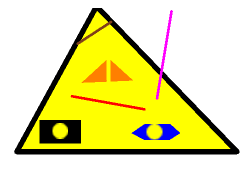 | 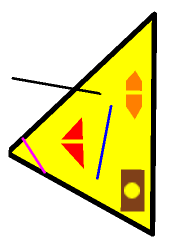 | | 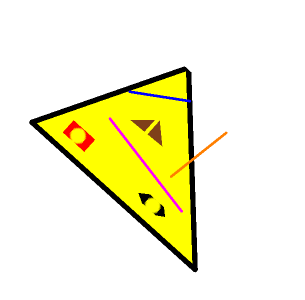 |

**Testing Stimuli**

| Examples | | |
| --- | --- | --- |
| 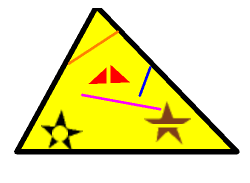 | 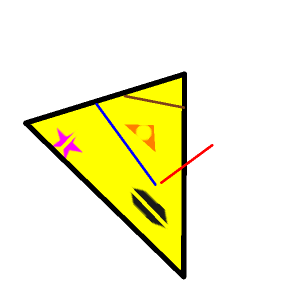 | 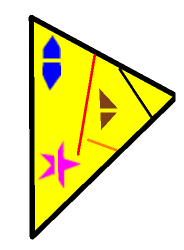 |
| 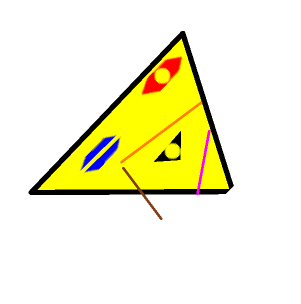 | 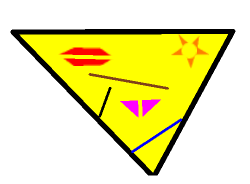 | 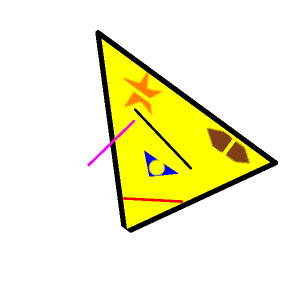 |
| 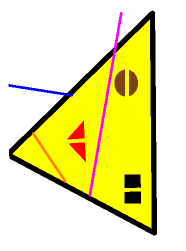 | 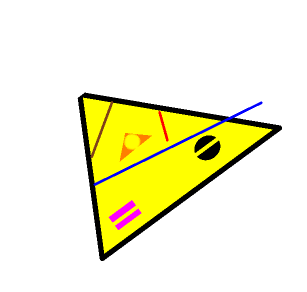 | 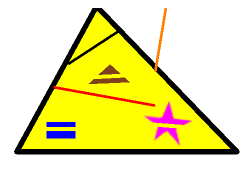 |
| 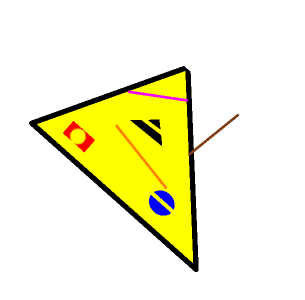 | 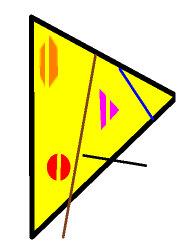 | 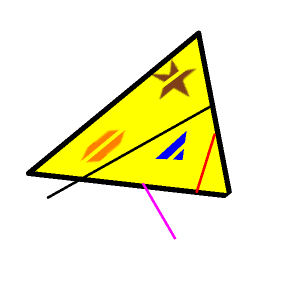 |
| Close-In Nonexamples Lacking Inside Line | | |
| 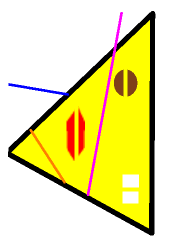 | 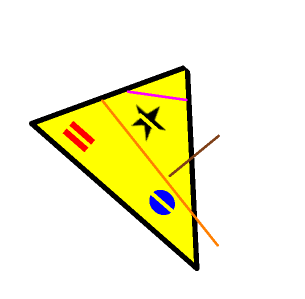 | 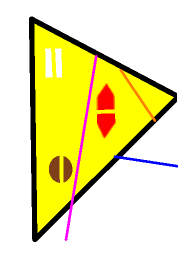 |
| 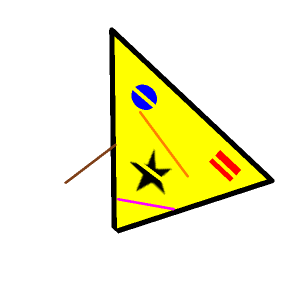 | 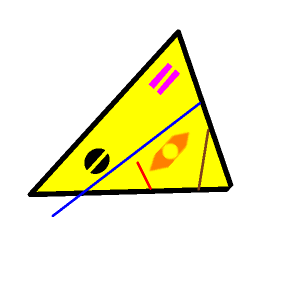 | 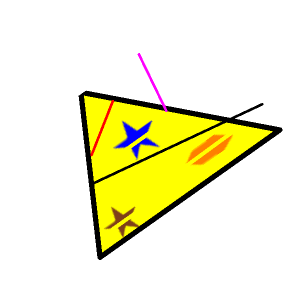 |
| Close-In Nonexamples Lacking Horizontal Line Filling Geometric Figure Closest To The Intersection Of The Two Longest Sides | | |
| 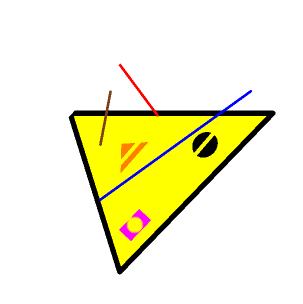 | 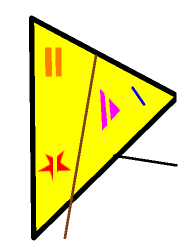 | 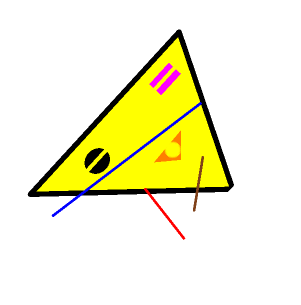 |
| 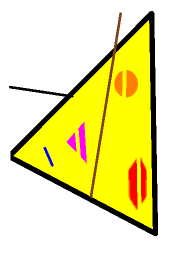 | 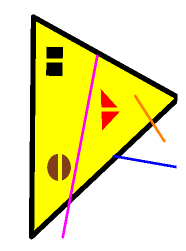 | 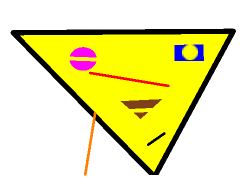 |
| Close-In Nonexamples Lacking Internal Triangle | | |
| 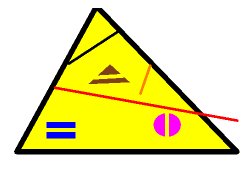 | 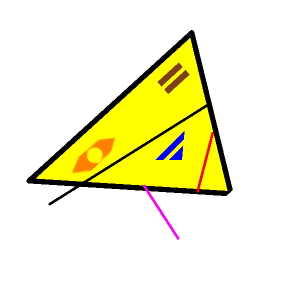 | 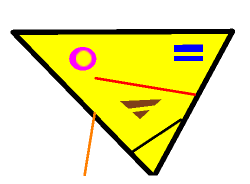 |
| 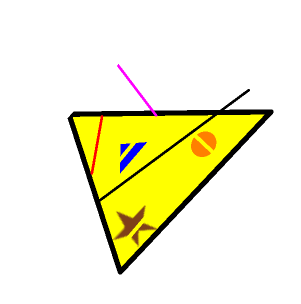 | 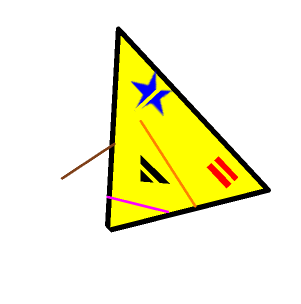 | 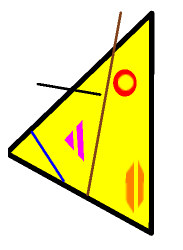 |
| Far-Out Nonexamples (lack all three features) | | |
| 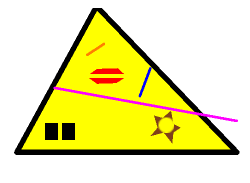 | 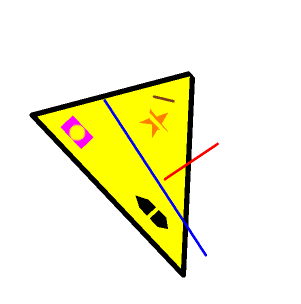 | 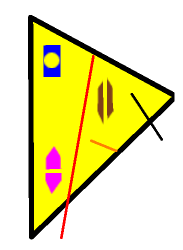 |
| 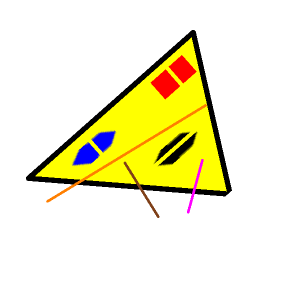 | 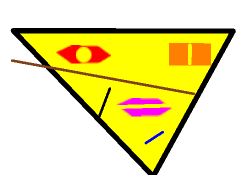 | 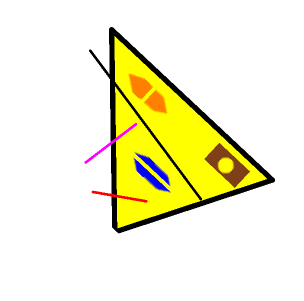 |
| 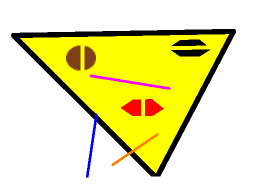 | 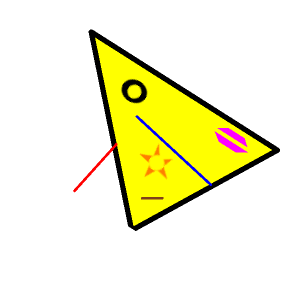 | 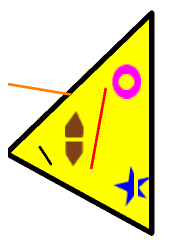 |
| 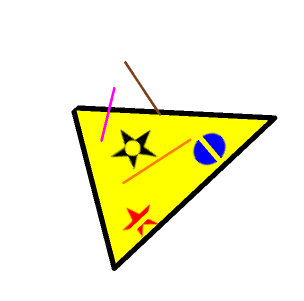 | 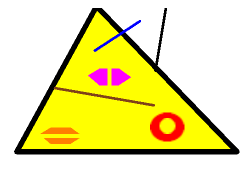 | 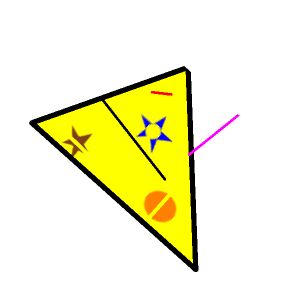 |
| 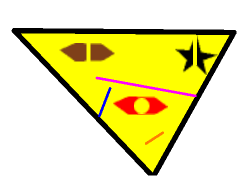 | 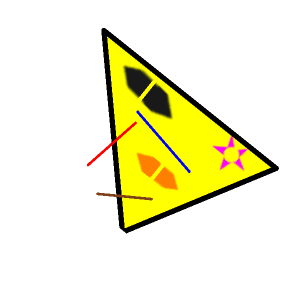 | 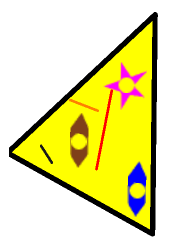 |
| 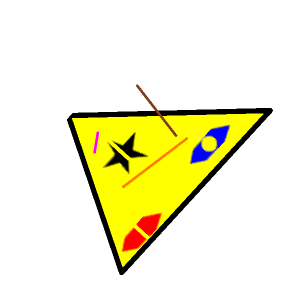 | 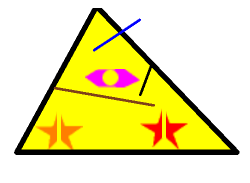 | 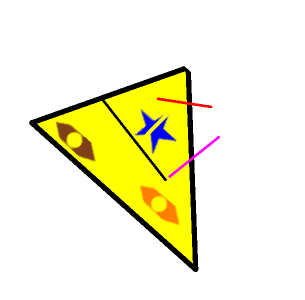 |
